# Supplementary material for: High throughput ANI analysis of 90K prokaryotic genomes reveals clear species boundaries
Source: Nat Commun. 2018 Nov 30;9:5114. doi: 10.1038/s41467-018-07641-9 (PMC6269478; doi:10.1038/s41467-018-07641-9)
Supplement: Supplementary file 1 — Supplementary Information [file 41467_2018_7641_MOESM1_ESM.pdf]

High Throughput ANI Analysis of 90K Prokaryotic Genomes Reveals Clear Species Boundaries

Jain *et al.*

Supplementary Table 1: Evaluation of FastANI accuracy and performance while varying the fragment length *l* used in the algorithm. We measured Pearson correlation coefficients of FastANI estimate with BLAST-based ANI computation (ANI_b) as well as runtime and memory usage for each value of fragment size (1 Kbp – 10 Kbp). This experiment was conducted using datasets D3 and D4. From the table, it is evident that increasing fragment size improves runtime and memory usage, but negatively affects accuracy. Based on these tradeoffs, we set the fragment size to 3 Kbp in the FastANI implementation.

**Dataset**	**Metric**	**Fragment size**			
		**1 Kbp**	**3 Kbp**	**5 Kbp**	**10 Kbp**
D3	Correlation with ANI_b	0.9980964	0.9952683	0.9919395	0.9867375
	Runtime (index phase) in seconds	2589.72	1666.58	1435.32	1145.05
	Runtime (compute phase) in seconds	4737.62	2099.08	1286.37	546.78
	Memory (GB)	113.75	48.35	28.95	14.73

D4 (without two outlier genome assemblies)	Correlation with ANI_b	0.9649292	0.9439152	0.9019418	0.7865688
	Runtime (index phase) in seconds	256.63	175.20	158.73	124.71
	Runtime (compute phase) in seconds	746.63	254.11	172.82	74.32
	Memory (GB)	12.59	4.38	3.16	1.59

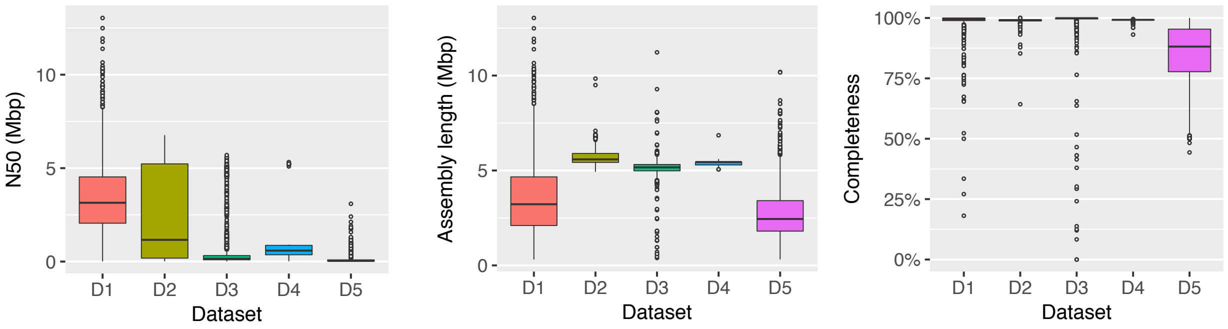

Supplementary Figure 1: N50, genome-length and completeness distribution for the five datasets D1-D5 is shown using boxplots. Genome completeness was estimated using the presence of marker genes in CheckM (v1.0.3) [1]. All five datasets exhibit different assembly N50 and length characteristics. We also note that majority of genomes in datasets D1-D4 are complete. Genomes in the D5 dataset have low completeness because all genomes in D5 were assembled using metagenomics.

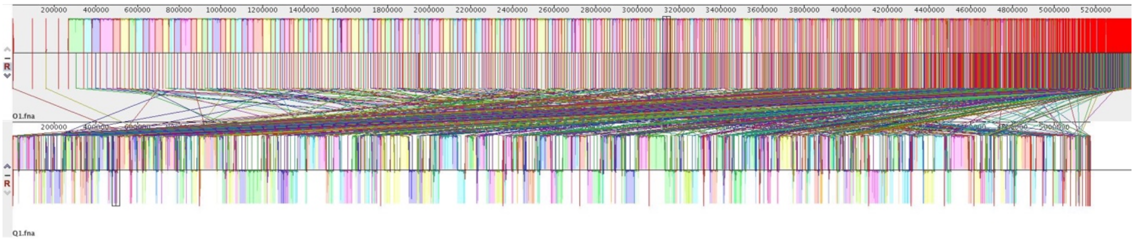

Mauve alignment of first outlier B. anthracis strain 2002734165 against the query strain

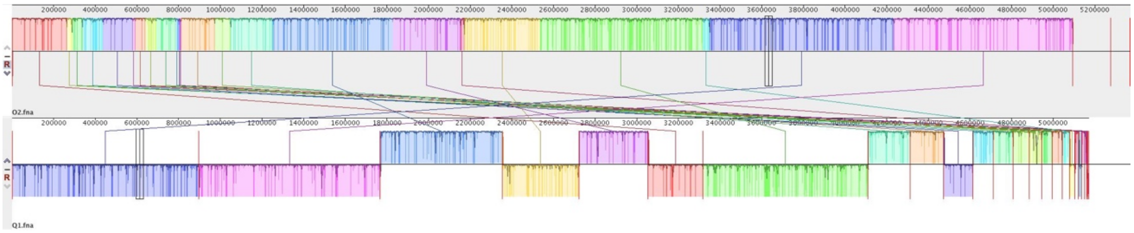

Mauve alignment of second outlier B. anthracis strain Ba_A2012_AAAC01000001 against the query strain

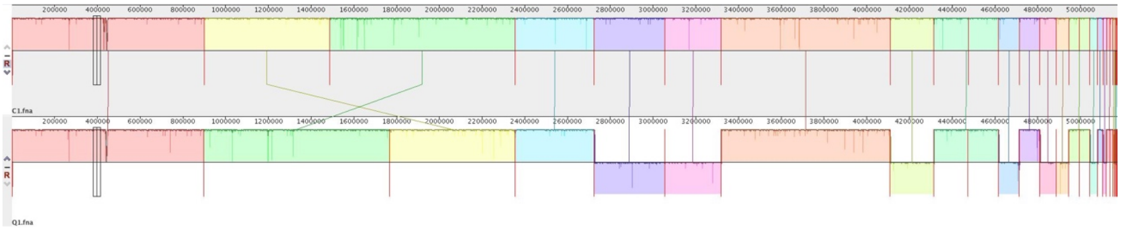

Mauve alignment of first randomly picked B. anthracis strain 2000031757 against the query strain

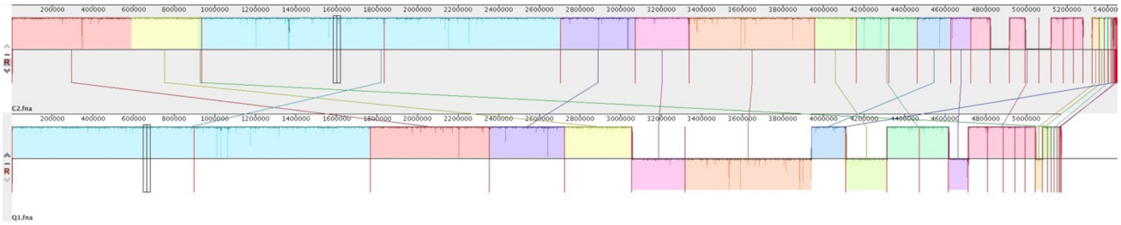

Mauve alignment of second randomly picked B. anthracis strain 2002734211 against the query strain

Supplementary Figure 2: Top two plots show the mauve alignments of the two outlier *B. anthracis* strains (2002734165 and Ba_A2012_AAAC01000001) against the query strain (2000031001) used in D4 dataset. Bottom two plots show the mauve alignments of two randomly picked *B. anthracis* strains against the query strain. The top two outlier strains show unusually higher degree of recombination and gaps than we expect between any two correctly sequenced and assembled *B. anthracis* strains. Same behavior was also observed using visualization support in FastANI software (figures not shown here).

CheckM [1] statistics for the first outlier genome indicated high strain heterogeneity (i.e., contamination from closely related taxa) of 9%. Mean strain heterogeneity in dataset D4 is 0.13%. Quality control and reassembly of raw sequences using Sickle [2] and Spades [3] respectively didn't improve the assembly quality, indicating contamination at the read level. Based on the CheckM estimates, the second outlier genome had the highest incompleteness of 7% in dataset D4. Reads for the second genome were not publicly accessible to perform a re-assembly.

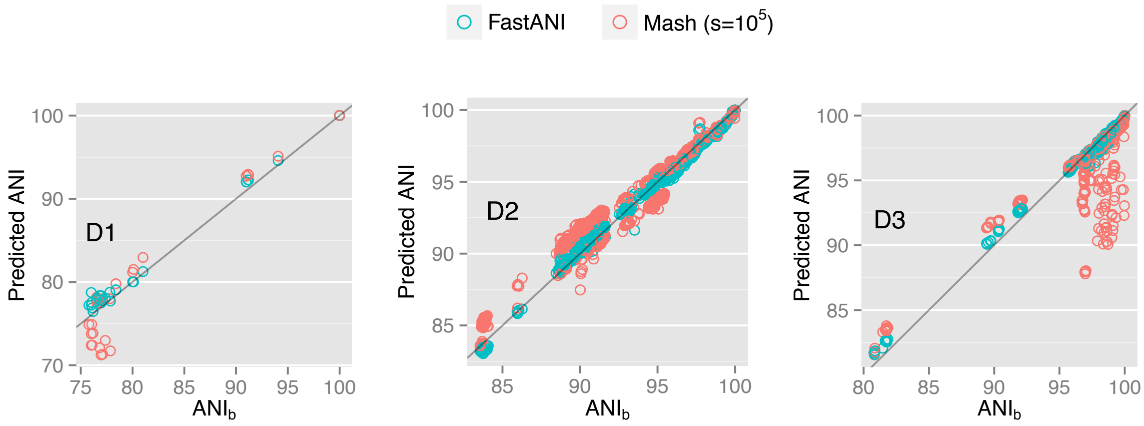

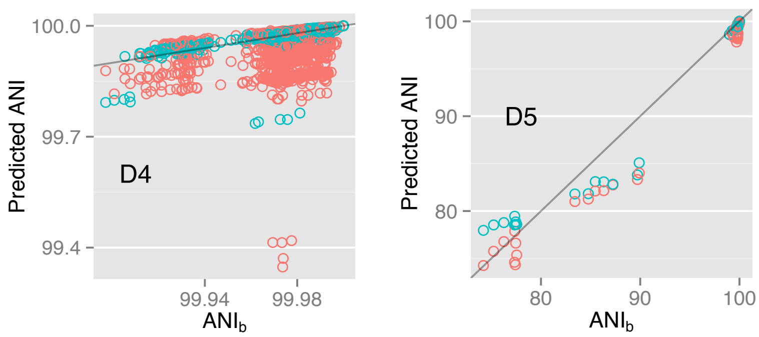

Pearson correlation with ANI_b		
	FastANI	Mash (s=10^5)
D1	**0.998**	0.973
D2	**0.997**	0.962
D3	**0.994**	0.936
D4	**0.720**	0.198
D5	0.981	**0.988**

Supplementary Figure 3: Correlation of FastANI and Mash output with ANI_b using the five datasets D1-D5 listed in Table 1. For a more robust comparison, we re-executed the experiment in main text (Fig. 1) with five randomly picked query genomes per dataset that were typically assigned to different species. Similar to what we observed before (Fig. 1, Table 2), FastANI continues to demonstrate either superior or competitive performance than Mash using all the datasets.

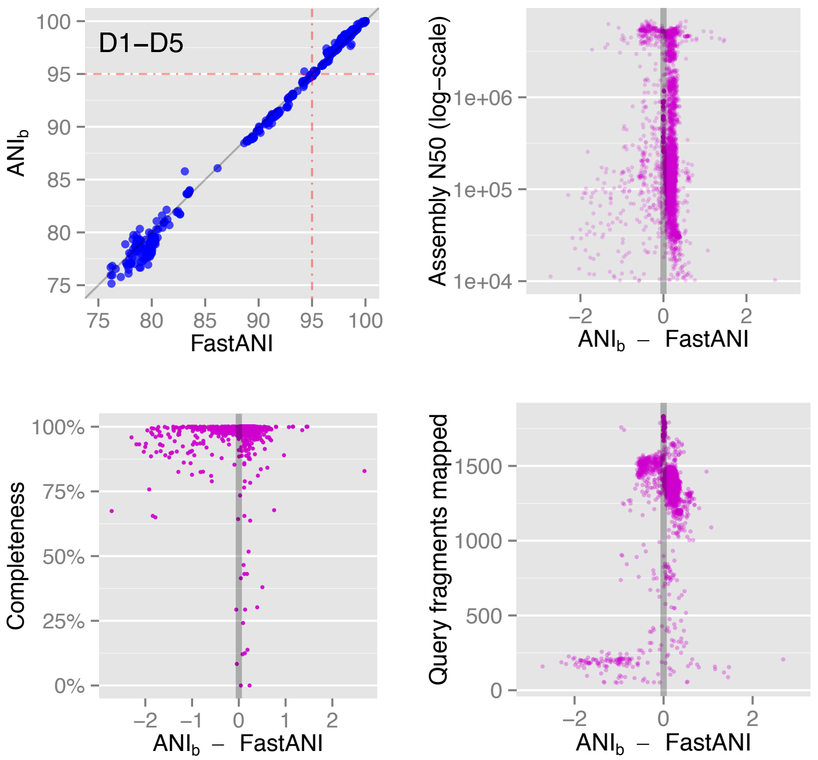

Supplementary Figure 4: FastANI's aggregate accuracy and error characteristics based on datasets D1-D5. Upper left plot shows the FastANI and ANI_b correlation. The remaining three plots show differences between FastANI and ANI_b value versus reference genome assembly quality (N50 and genome completeness) and the number of reciprocal fragments that matched between query and reference genome for each comparison. Genome completeness was estimated using CheckM (v1.0.3) [1]. Difference in the computed ANI values is relatively higher when there are few reciprocal fragments as shown in bottom-right plot, which typically happens for distant genomes (i.e., ANI close to 80%). Overall, these results show no significant biases associated with these factors.

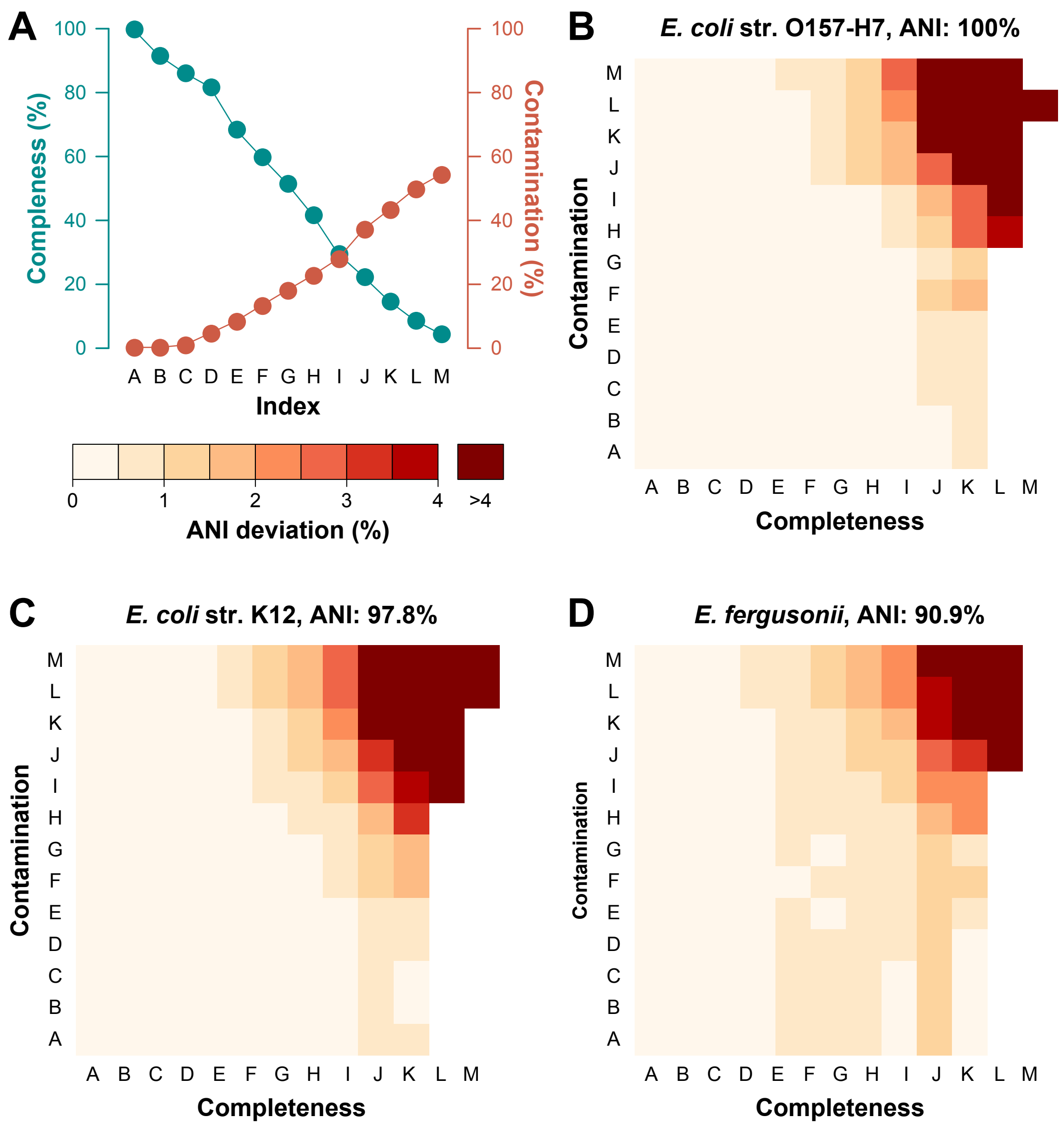

Supplementary Figure 5: We measure the effect of genome completeness and contamination on FastANI's accuracy using simulation. The completeness and contamination were simulated by manipulating the read composition of *E. coli* str. O157-H7 prior to its assembly, i.e., under-sampling the reads at various scales to induce incompleteness and adding reads of *Pseudomonads aeruginosa* str. PAO1 to induce contamination. Degrees of completeness and contamination were measured using the gene composition of assembled genome (see Panel A). Levels A to M indicate decreasing genome completeness and increasing contamination. For each combination of completeness and contamination, FastANI's accuracy with respect to BLAST-based ANI (ANI_b) is presented. Panels B-D show FastANI's deviation from ANI_b when computing ANI between the simulated *E. coli* str. O157-H7 genome against the reference genomes of *E. coli* str. O157-H7 genome (ANI=100%), *E. coli* str. K12 genome (ANI=97.8%) and *E. fergusonii* genome (ANI=90.9%) respectively. Missing (blank) values are those that FastANI failed to estimate due to insufficient hits.

Results show that the contamination had almost no effect at all on FastANI's output quality, except when there was also a very low completeness. Also, when completeness was >50% there was basically no effect, but around 20% completeness and below, the estimate became unreliable which is likely true for BLAST-based ANI as well.

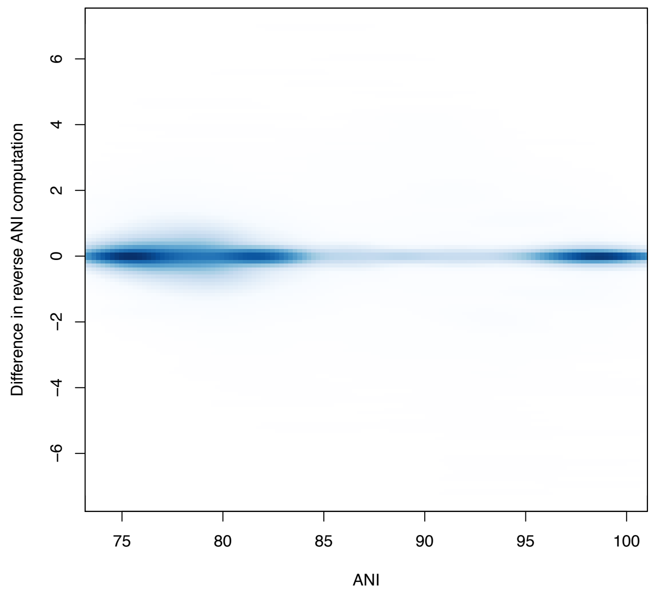

a) *Smooth scatter plot reflecting the effect of changing input order of genome pairs on FastANI. Y-axis shows the difference caused due to changing the input order and x-axis shows the ANI value obtained from FastANI. Ideally values on y-axis should equal zero. This data was obtained from FastANI run on the set of 89,499 genomes. FastANI reported 451 million genome pairs in the above ANI range.*

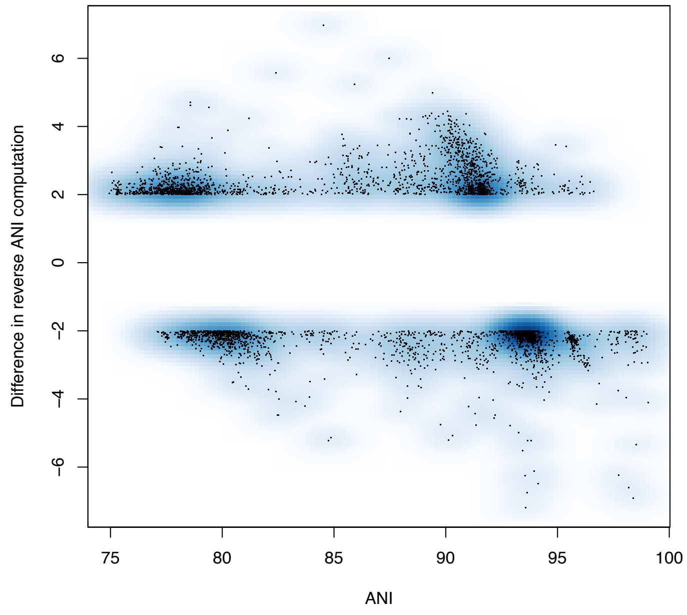

b) 4,966 genome pairs out of 451 million (0.001%) show a difference greater than 2 on changing their input order to FastANI. Above plot explicitly shows these outlier genome pairs.

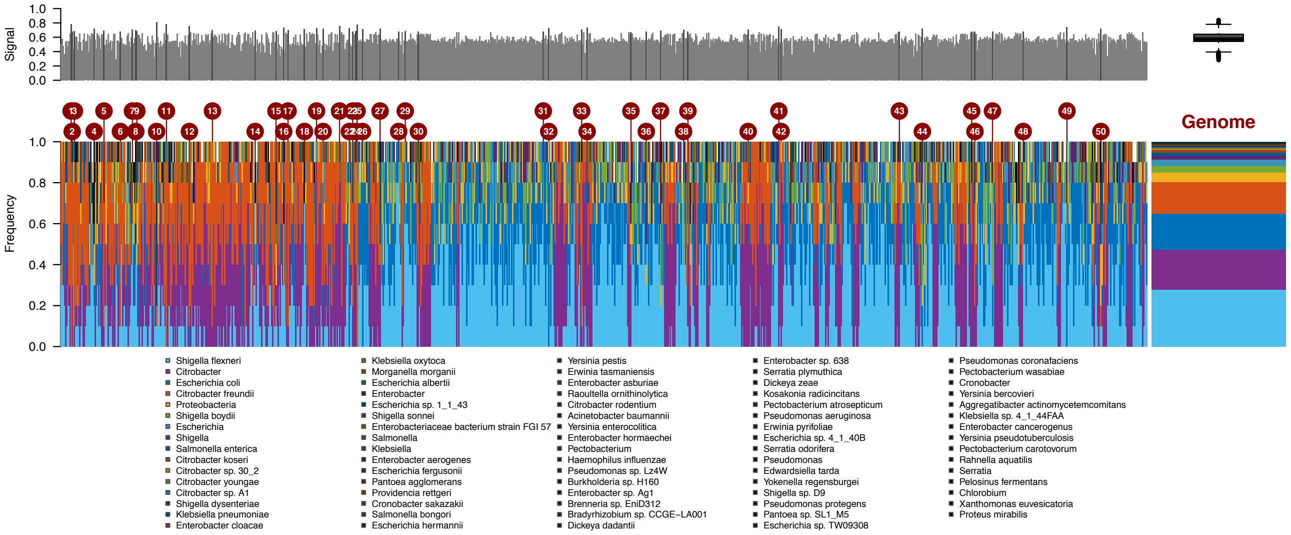

c) MyTaxaScan [4] analysis of Shigella Flexneri strain 1235_66_GCA_000268065 draft genome assembly. Top two contamination sources are species of the Citrobacter genus and E. coli genomes.

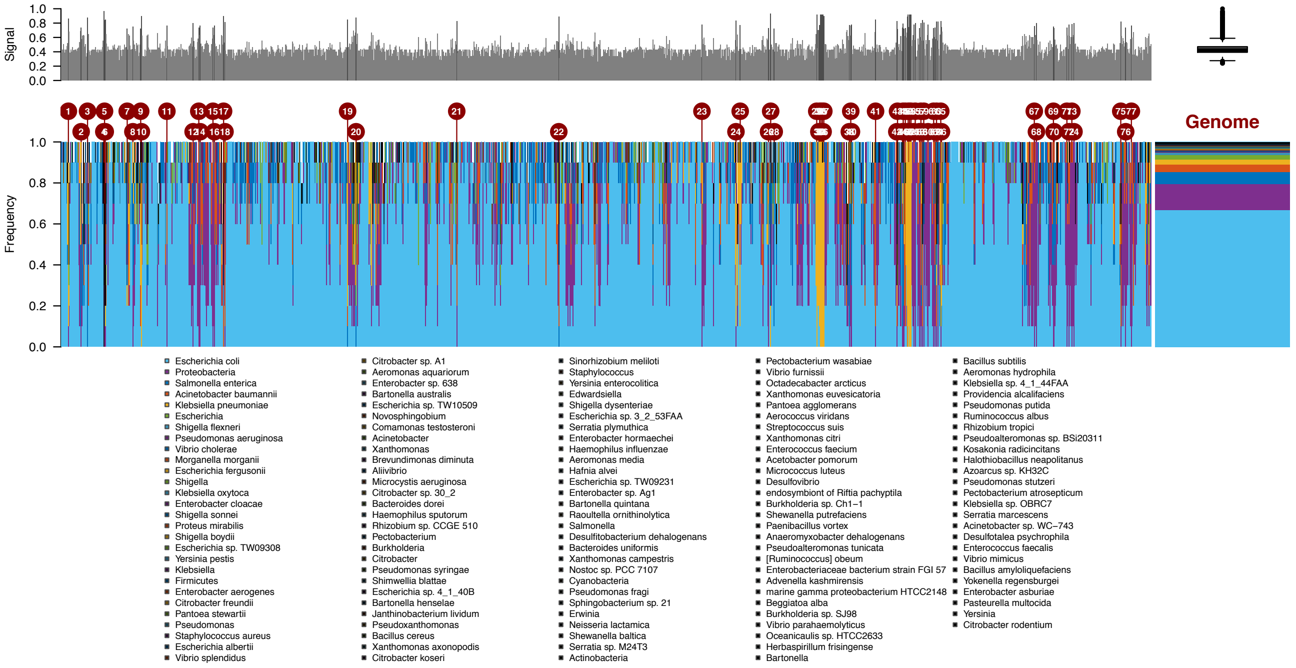

d) MyTaxaScan analysis of Escherichia coli strain NC_011752 draft genome assembly. Top two contamination sources are species in Proteobacteria phylum and S. enterica genome.

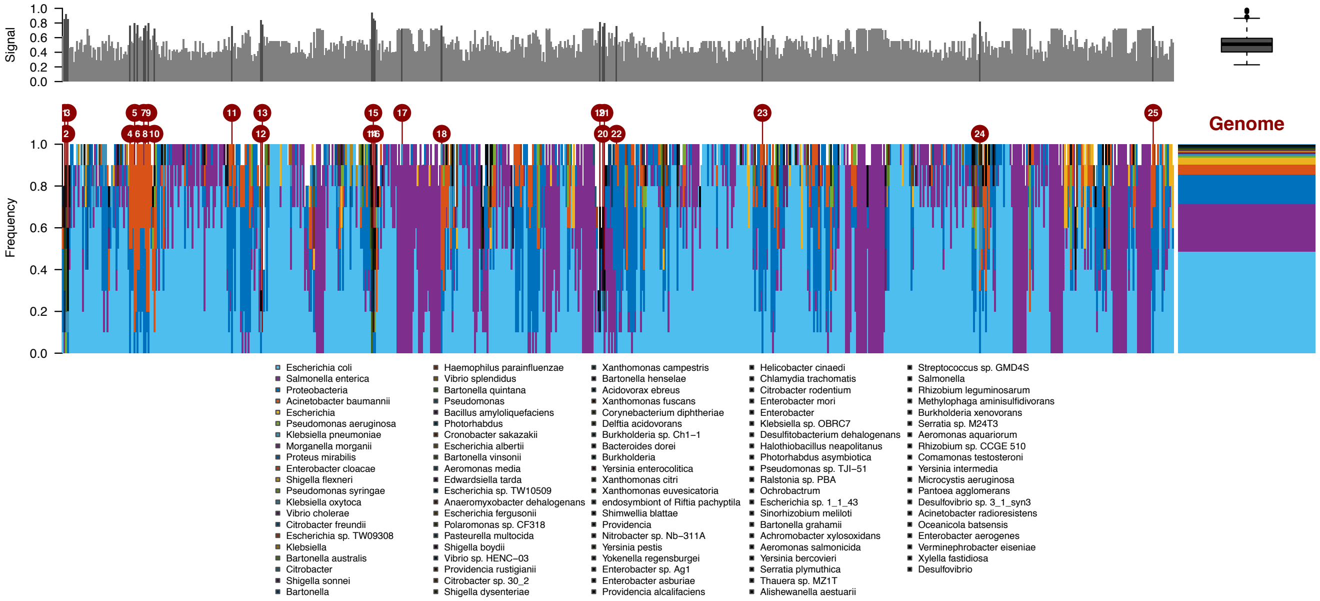

e) MyTaxaScan analysis of Salmonella enterica strain NC_010716 draft genome assembly. Top two contamination sources are from E. coli genomes and other members of the Proteobacteria phylum.

Supplementary Figure 6: FastANI accepts genome pairs <reference genome, query genome> as input. For most genome pairs, input order caused an insignificant change in the FastANI tool's ANI estimate. We first demonstrate this by showing a smooth-scatter plot of 451M genome pairs [plot a]. Among them, we observed 4,966 outlier genome pairs (0.001% of 451M) that show difference ≥ 2 [plot b].

On further investigating these outliers, we concluded that they are caused by highly contaminated genome assemblies. We used MyTaxaScan [4] to perform quality check of the top three genomes that contribute to the 4,966 outliers: a) *Shigella flexneri* strain 1235_66_GCA_000268065 assembly (part of 1,941 outliers), b) *Escherichia coli* strain NC_011752 assembly (part of 632 outliers), and c) *Salmonella enterica* strain NC_010716 assembly (part of 505 outliers) [plots c-e]. To generate the MyTaxaScan plots, we divided each genome in windows of 10 genes, and estimated the taxonomic profile for each window using MyTaxa [5]. The resulting most-likely classification is represented as stacked bars per window, and the profile generated from all genes in the genome is presented on the right-hand side (see corresponding legends for colors). We compared the distribution resulting from each window against the genome-wide distribution using Hellinger distances, represented as grey bars in the top panel of each plot, and highlight regions with abnormally high distances (numbered red pinheads). Multiple colored peaks in all the three MyTaxaScan [4] plots highlight significant contamination in these assemblies from other species.

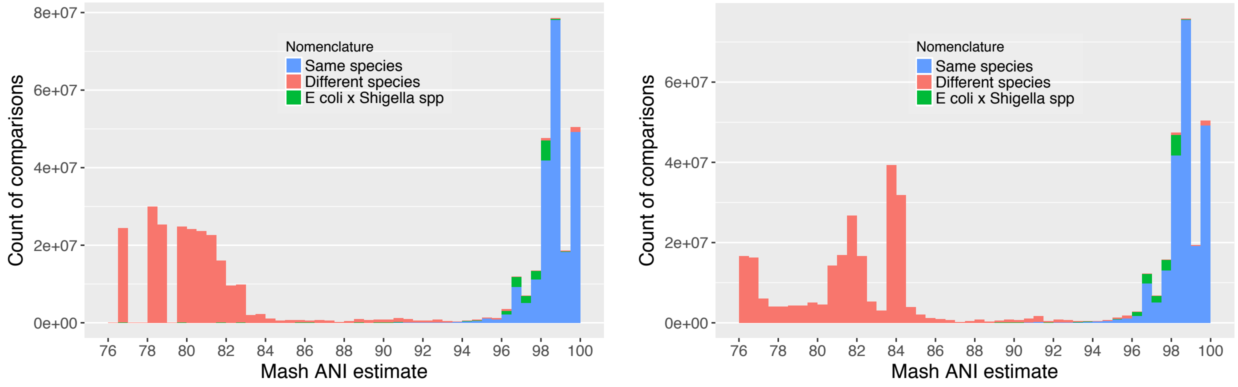

a) Mash sketch size = 1,000

b) Mash sketch size = 10,000

Supplementary Figure 7: Histogram plot showing the distribution of ANI values among the 90K genomes with ANI estimated using Mash. Similar to the FastANI results presented in main text (Fig. 3c), the bimodal ANI distribution is persistent. Moreover, the 95% ANI species cutoff is evident using Mash results as well. Considering the shapes of the two peaks, the right peak matches with the one obtained using FastANI, but shape of the left peak differs. In lower identity range however, we expect FastANI's output (Fig. 3c) to be more reliable than Mash.

For this all vs. all comparison, Mash took much less time compared to FastANI- only 51 CPU hours and 359 CPU hours with sketch sizes 10^3 and 10^4 respectively. However, Mash failed to produce output with sketch size 10^5 due to a runtime error.

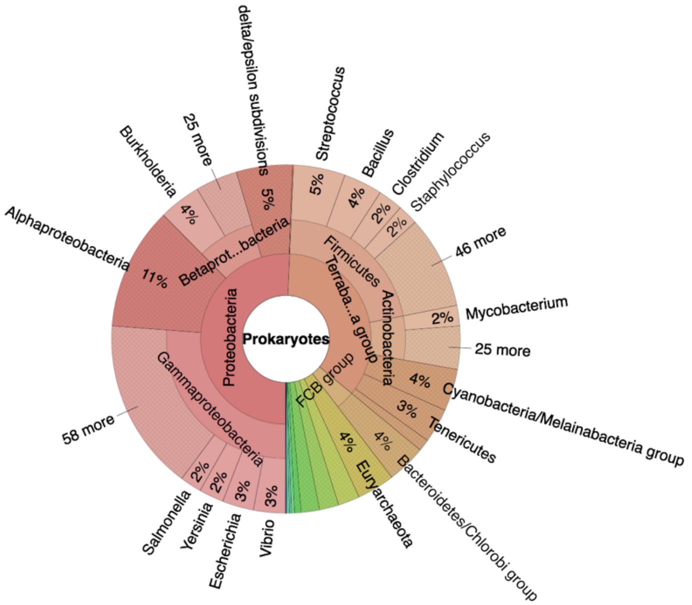

Composition of first 1000 prokaryotic genomes in NCBI database by their genus assignment (11/1985 – 02/2008)

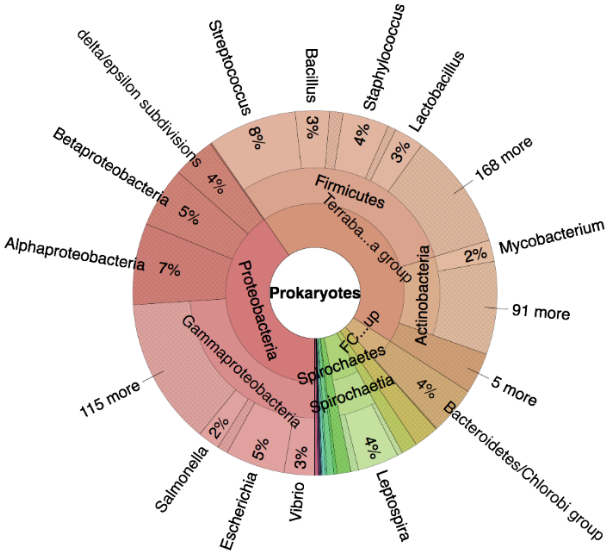

Composition of first 5,000 prokaryotic genomes in NCBI database by their genus assignment (11/1985 – 02/2012)

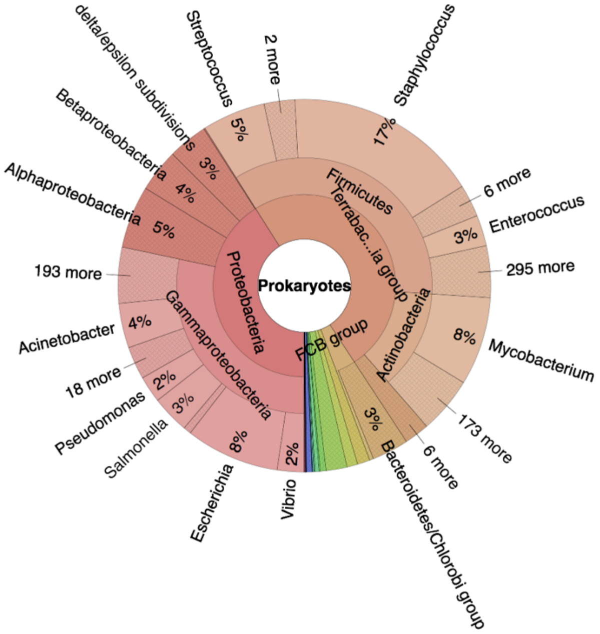

Composition of first 25,000 prokaryotic genomes in NCBI database by their genus assignment (11/1985 – 05/2014)

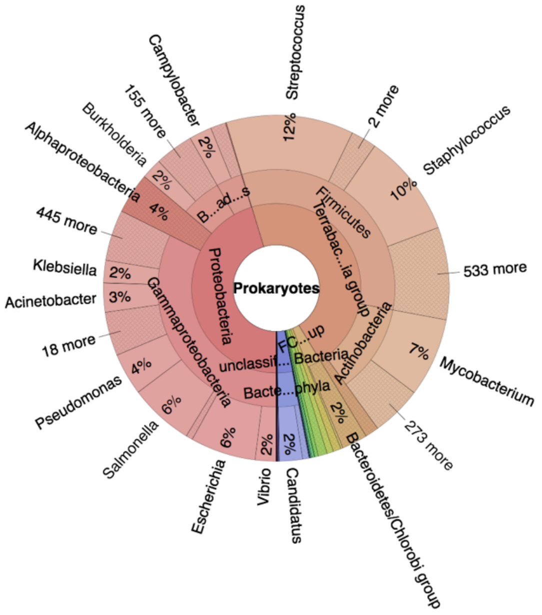

Composition of all 89,499 prokaryotic genomes in NCBI database by their genus assignment (11/1985 – 03/2017)

Supplementary Figure 8: Composition of draft prokaryotic assemblies in the NCBI database with time at the genus level is visualized using Krona [6] charts. As expected, genera of health or biotechnological importance have dominated the database progressively. For each of these cohorts, ANI distribution density curves are shown in Fig. 3b.

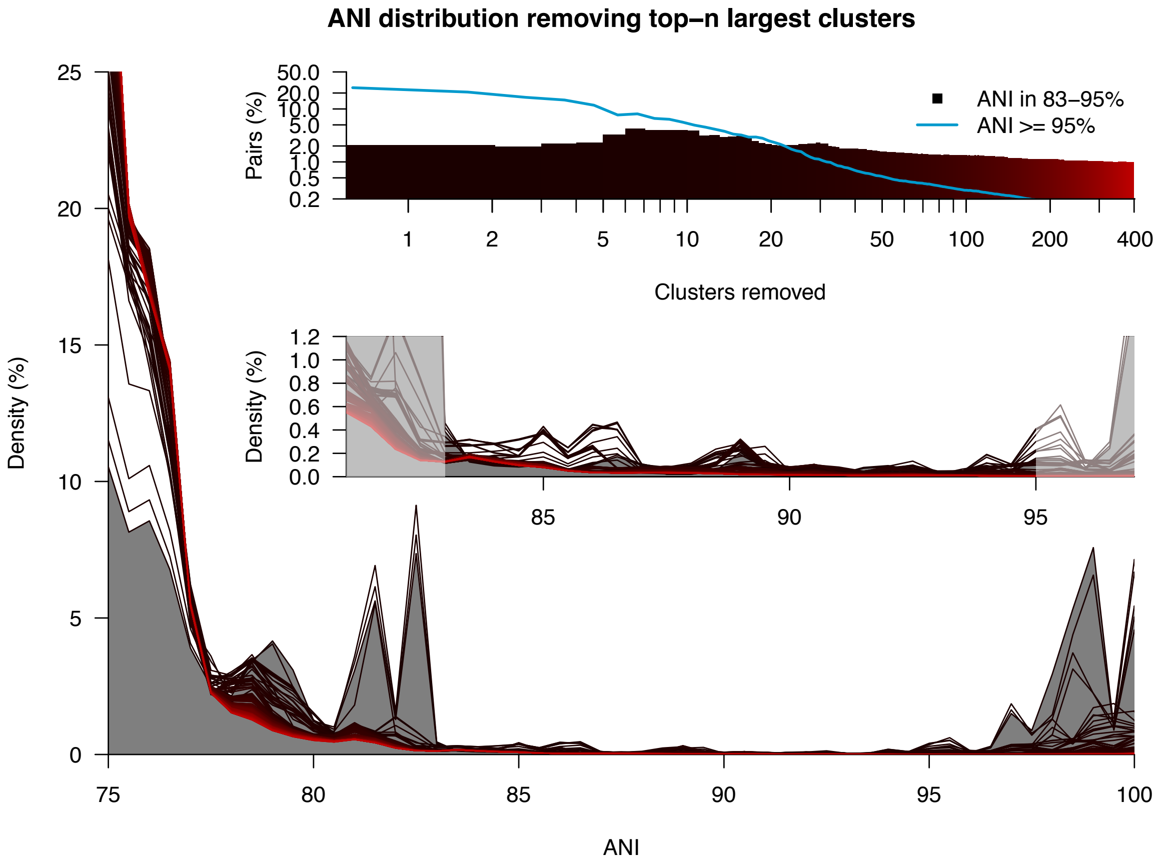

Supplementary Figure 9: The sequence discontinuity was also evident when the species with large numbers of representatives in the database were iteratively removed from the database. We computed genome clusters corresponding to genome pairs with ≥95% ANI values. The main plot shows a histogram of ANI values (filled grey) for all genomes in the database (same as Fig. 3a). The different lines shown correspond to the resulting histograms after removing the top-n largest clusters, with n ranging from zero (black line) to 400 (brightest red line). Notice that the second peak on the right side starts to disappear when all 400 clusters are removed due to reduced intra-species comparisons in the database. The top inset shows how the fraction of pairs that fall into the 83-95% ANI region (with respect to the 75%-100% ANI region) vary with the number of clusters removed (black-to-red filled area, both in log scale), as well as the fraction of pairs above 95% (blue line). Notice that while removing the top-n largest clusters, the fraction of pairs inside the valley is consistently below 5%. The bottom inset shows a magnification of the valley region (83% - 95% ANI).

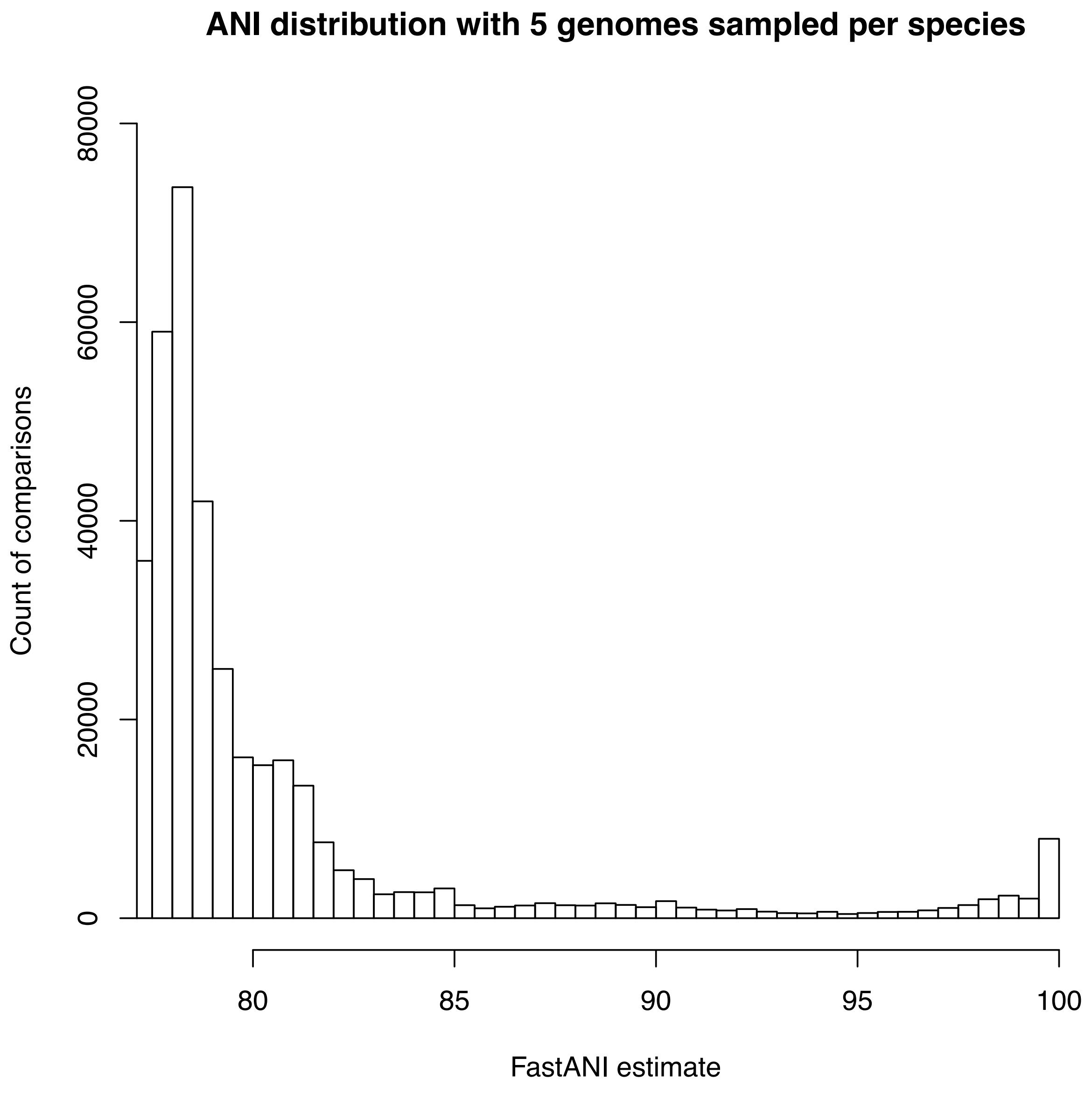

Supplementary Figure 10: Distribution of pairwise ANI values in a genome set that is characterized by equal representation of named species in our dataset. First, we selected all named species for which there were five or more genomes available in the NCBI database (750 in count). A custom database was created with five genomes randomly picked for each named species, yielding a total of 3,750 genomes. Discontinuity is still evident with FastANI reporting only 0.2% inter-species pairs in the valley region (83%-95%) out of the total inter-species pair count present in the sampled set. The right-hand side peak is small (relative to Fig. 3a) because we have only five genomes per species yielding only 18,750 intra-species pairs in the sampled set. Similar observations were drawn when we sampled two genomes per species yielding a set of 4,838 genomes (2,419 species x 2) [data not shown].

References

1. Parks, Donovan H., et al. "CheckM: assessing the quality of microbial genomes recovered from isolates, single cells, and metagenomes." *Genome research* 25.7 (2015): 1043-1055.
2. Joshi et al. “Sickle: A sliding-window, adaptive, quality-based trimming tool for FastQ files”. URL: https://github.com/najoshi/sickle (Version 1.33)
3. Bankevich, Anton, et al. "SPAdes: a new genome assembly algorithm and its applications to single-cell sequencing." *Journal of computational biology* 19.5 (2012): 455-477.
4. Rodriguez-R et al. “Microbial Genomes Atlas: Standardizing genomic and metagenomic analyses for Archaea and Bacteria”. URL: http://microbial-genomes.org/
5. Luo, Chengwei, Luis M. Rodriguez-r, and Konstantinos T. Konstantinidis. “MyTaxa: an advanced taxonomic classifier for genomic and metagenomic sequences.” *Nucleic acids research* 42.8 (2014): e73-e73.
6. Ondov BD, Bergman NH, and Phillippy AM. “Interactive metagenomic visualization in a Web browser”. BMC Bioinformatics. 2011 Sep 30; 12(1):385.
